# Genetic Material Manipulation and Modification by Optical Trapping and Nanosurgery-A Perspective

**DOI:** 10.3389/fbioe.2020.580937

**Published:** 2020-09-18

**Authors:** Alfonso Blázquez-Castro, José Fernández-Piqueras, Javier Santos

**Affiliations:** ^1^Department of Biology, Faculty of Sciences, Autonomous University of Madrid, Madrid, Spain; ^2^Genome Dynamics and Function Program, Genome Decoding Unit, Severo Ochoa Molecular Biology Center (CBMSO), CSIC-Autonomous University of Madrid, Madrid, Spain; ^3^Institute of Health Research Jiménez Diaz Foundation, Madrid, Spain; ^4^Consortium for Biomedical Research in Rare Diseases (CIBERER), Carlos III Institute of Health, Madrid, Spain

**Keywords:** optical trapping, optical tweezers, laser scissors, genetic manipulation, cell surgery, genomic instability, cytogenetics, DNA damage response

## Abstract

Light can be employed as a tool to alter and manipulate matter in many ways. An example has been the implementation of optical trapping, the so called optical tweezers, in which light can hold and move small objects with 3D control. Of interest for the Life Sciences and Biotechnology is the fact that biological objects in the size range from tens of nanometers to hundreds of microns can be precisely manipulated through this technology. In particular, it has been shown possible to optically trap and move genetic material (DNA and chromatin) using optical tweezers. Also, these biological entities can be severed, rearranged and reconstructed by the combined use of laser scissors and optical tweezers. In this review, the background, current state and future possibilities of optical tweezers and laser scissors to manipulate, rearrange and alter genetic material (DNA, chromatin and chromosomes) will be presented. Sources of undesirable effects by the optical procedure and measures to avoid them will be discussed. In addition, first tentative approaches at cellular-level genetic and organelle surgery, in which genetic material or DNA-carrying organelles are extracted out or introduced into cells, will be presented.

## Introduction

The judicious manipulation of light and optical properties has allowed the development of many uses and applications in the field of Biology and Biotechnology. Perhaps one of the most obvious is its use in optical microscopy, which paved the way to discover and study the microscopic world. Optical applications also provide ways for manipulating biological entities and structures, by making use of different techniques like photochemistry, microirradiation and optogenetics, to name only a few. For the last three centuries techniques have been available to observe microscopic biological samples. The active manipulation of microscopic objects with light has been possible since the early years of the 20th Century. However, the sources providing light were incoherent and inherently flawed to achieve certain goals, like achieving true (sub)-micrometric scale of action or providing monochromacity or very short pulsed action. This changed on May 1960, when Theodore Maiman produced the first amplified light pulses through stimulated emission of optical radiation. The public announcement was made a few months later, in August 1960 (Maiman, 1960). The new light source was known as the laser, from light amplification by stimulated emission of radiation. As related to this review, the light output from a laser displays adequate features to implement optical tweezers and controlled photoablation in microscopic biological systems, like cells or cellular structures. Thus, this review focuses on the uses and applications of optical tweezers (OT) and laser scissors (LS) for the study and manipulation of genetic material.

Laser light displays some very particular features that make it the ideal light source for OT and LS like coherence, very small divergence, continuous or pulsed output, or light monochromacity. Coherence and small divergence make it possible for extremely tight light focusing, which can translate to huge photon fluxes across microscopic spots. These features were considered by Arthur Ashkin when he proposed a new kind of optical actuator, one capable of moving or holding microscopic structures by making use of focused light (Ashkin, 1970). This new tool came to be known as an optical tweezers. The topic of OT is very broad and its working fundamentals quite beyond the scope of this review. Many reviews (Berns et al., 1997; Bowman and Padgett, 2013; Gao et al., 2017; Dhakal and Lakshminarayanan, 2018) on the mechanisms and multiple applications have been written along the years and can be consulted by those interested. Here, we will focus on the use of OT to manipulate and study genetic material and structures. Similar arguments can be said about laser ablation, the process permitting LS. The topic is very broad and here we are only concerned with its application to genetic materials. Readers interested in the general principles can consult some reviews (Vogel and Venugopalan, 2003; Berns et al., 1997; Ronchi et al., 2012; Vasa and Mathur, 2016).

**FIGURE 1:**
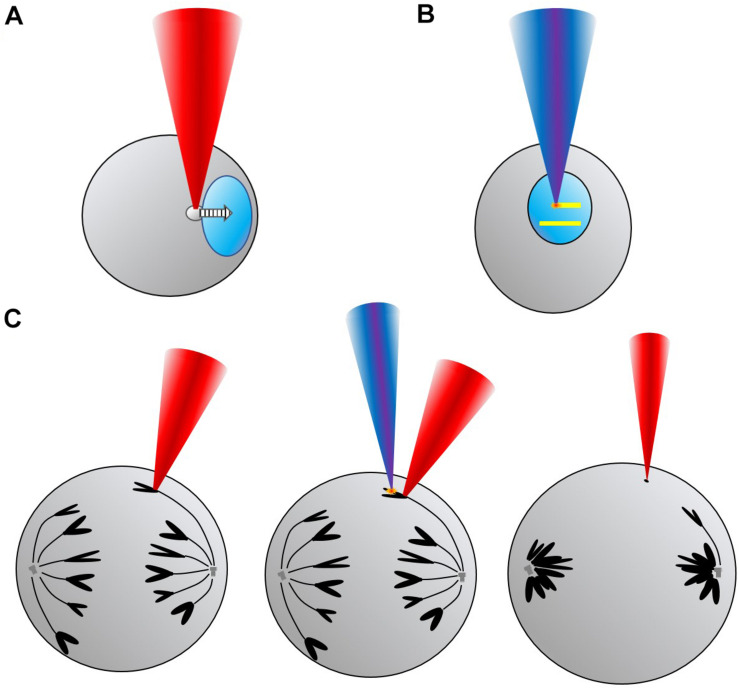
Conceptual scheme showing some of the uses and applications of laser scissors (LS) and optical tweezers (OT) for manipulation and modification of genetic material in different formats. **(A)** Top left, translation of a cell nucleus (light blue) toward the right by trapping a lipid granule (gray) with OT (red beam). **(B)** Top right, direct production of DNA damage (yellow lines) by scanning a LS (deep blue beam) across the nucleus to study, for example, DNA repair mechanisms. **(C)** Bottom, sequence showing OT hold of one anaphase chromosome (black), followed by microablation (orange “spark”) with LS of one of the chromosome arms, and, finally, trapping of the chromosome fragment (black dot) with OT while the cell enters telophase.

This review will present some of the more relevant studies and experiments in the field of Genetics, Cytogenetics and Cell Biology which have employed OT and/or LS to manipulate or modify genetic materials and structures (Figure 1). For example, it is possible to move intracellular organelles, like the nucleus (Figure 1A), with OT, or induce localized genetic damage with sub-micrometric precision by means of LS (Figure 1B). Combining both OT and LS intracellular cuts of selected structures and trapping of the produced fragments (e.g., from chromosomes) can be accomplished (Figure 1C). By genetic material we broadly refer to nucleic acid molecules (DNA and RNA), chromatin and chromosomes. But perturbation of cell structures such as the nucleoli, the cell nucleus or organelles (e.g., mitochondria), deemed very relevant for the topic at discussion due to their relation to the genetic material of the cell, will also be introduced. First, a brief historical survey of the initial experiments in the field will be provided. Historically, laser ablation was introduced earlier than OT to modify genetic material. Therefore, the fundamentals and experiments making use of LS will be presented first. Use of OT in the biological field took place in the late 1980s. Relevant results will then be presented. Then, experiments taking advantage of the combination of OT plus LS will be introduced, showing the range of possibilities these two techniques offer when employed together. Finally, a section dedicated to potential developments and unexplored avenues in the field will be provided as perspectives. The review will finish with some conclusions.

The research field of optical manipulation and alteration of biological structures is very broad at present. In consequence some topics have been purposefully left outside the specific contents of this review for the sake of space. A very recent review, specifically focused on the use of OT and LS for the study of chromosomes, has been published in this special issue on Optical Trapping (Laser Tweezers) and Nanosurgery (Laser Scissors) (see Berns, 2020). Additional information complementing our work can be found in that authoritative publication. Closely related to the use of OT and LS in chromosomes is the study of their cellular movements and the mechanisms coordinating their distribution during cell division (mitosis and meiosis). Due to the very large amount of published information this is also is considered outside the strict scope of this review (see Berns et al., 2006; Heisterkamp et al., 2007; Khatibzadeh et al., 2014; Maiato et al., 2017; Oriola et al., 2018 for more information on this). Nevertheless, some hints will be provided given its relevance. Another interesting topic, the study of pure DNA mechanical properties (Heller et al., 2014) is excluded from this work, as it deals with nucleic acids properties that are not necessarily relevant in living biological cells.

The technique of cellular optoporation, widely employed to force cells to uptake compounds of interest (including nucleic acids) by means of pulsed laser light, was considered to be included in this review for some time. However, after critically pondering the matter, it was considered too broad to be included here. A relevant argument for excluding optoporation is because the action mechanism underlying the principle of optoporation works for many types of compounds, not just genetic material. Additionally, it was considered to be a procedure to passively introduce genetic material into a cell, but not a tool to actively manipulate or alter such material. Nevertheless, it remains a very important transfection technique. As such the interested reader can find adequate sources on optoporation in Xiong et al. (2016), Stewart et al. (2018), and Schneckenburger (2019). Other topics, with connections to the genetic manipulation by OT and LS, such as nuclear transplant and secondary oocyte manipulation in the field of artificial fertilization, fall outside the scope of this work, as these techniques mainly employ mechanical tools over optical procedures to achieve their goals.

## Historical Background

The use of light to microscopically alter biological structures and living organisms can be traced back to the works of Serge Tchakhotine (Berns et al., 1997). In the early 20th Century he published results on the effects of ultraviolet (UV) light on cells. However, incoherent UV light sources were employed which preclude optimum optical focusing and probably required dedicated optics to allow for adequate light transmission. As mentioned in the Introduction, the laser is the tool that has allowed for successful and reproducible biological microirradiation and ablation research. Charles Townes, one of the discoverers of stimulated emission of radiation and the maser/laser, discussed potential applications and effects of the new laser in Biology (Townes, 1962). He foresaw the laser as a source of pulsed photothermal action, non-linear photochemical excitation and microscopic ionization (plasma generation). He advanced *“.microsurgery include heating a particular part of a chromosome with the hope of causing specific mutations, disturbance of or destruction of a particular section of chromosome.*”, in relation to laser alterations of genetic material (chromosome) within living cells. Microirradiation experimental results over the years have proved him right in many of these early proposals.

The only available visible laser at the very early 1960s was the ruby laser. Bessis et al. (1962) first made use of this laser to irradiate subcellular structures in 1962. The next year, a pulsed ruby laser was employed to microirradiate *Spirogyra* cells at different subcellular structures, including the nucleus (Saks and Roth, 1963). Sometimes “dyeing” with methylene blue was necessary to damage certain structures at low output energies to precisely confine the effects. The authors stated that cytogenetics could be one of the research areas most benefited by this new laser technology. From this point on more and more works were published, reporting on an increasing number of (sub)-cellular structures being microirradiated: whole cell/plasma membrane micropuncturing (Bessis and Ter-Pogossian, 1965), nucleus and cytoplasm (Saks et al., 1965), mitochondria (Amy and Storb, 1965), mammal blastomeres (Daniel and Takahashi, 1965), and chromosomes (Berns et al., 1969a,b).

In spite of the initial success, the first lasers employed for cellular and subcellular irradiation lacked, in general, the adequate optical parameters (pulsed power, pulse length, irradiance, wavelength, etc.) to perturb the biological sample adequately. For this reason, many experiments during this early period had to made use of some kind of sample dyeing (e.g., methylene blue, janus green B or acridine orange) to sensitize the biological structure of interest to the employed laser’s spectral output (Saks and Roth, 1963; Amy and Storb, 1965; Bessis and Ter-Pogossian, 1965; Daniel and Takahashi, 1965; Berns et al., 1969a,b, 1976). It was necessary to increase the light absorption of the sample to initiate photochemical reactions. This was, probably, to the relatively small irradiances of the first laser systems (10^4^–10^6^ W cm^–2^) which, in combination with their visible (green, red) emission wavelength, proved inefficient to initiate non-linear photochemistry and/or plasma generation. As output energies increased and light pulses became shorter in the following years, it was unnecessary to increase sample absorption to induce the desired optical effects. From 1970 onward, as laser experimental procedures became less demanding, the number of publications reporting on laser microirradiation and ablation of cellular structures steadily grew. It is beyond this review to provide a detailed historical account, but there are texts providing an adequate introduction to the topic narrated by those who undertook this pioneer research (Berns, 1978, 2007a; Berns et al., 1981; Weber and Greulich, 1992).

On stark contrast to LS, it took much longer for OT to be introduced into biological research since the invention of the laser. OT were conceived in 1970 by Arthur Ashkin (1970). However, it took almost two decades for Ashkin himself to propose that optical gradients could be employed to manipulate biological structures (Ashkin and Dziedzic, 1987, 1989; Ashkin, 1991). Ashkin did not only make the theoretical proposal. He provided the first experimental proof of the OT procedure by successfully trapping tobacco mosaic virus particles, some unidentified mobile bacteria, living *E. coli*, yeast (*S. cerevisiae*), *Spirogyra* colonies, human erythrocytes, and different kinds of protozoa (Ashkin and Dziedzic, 1987; Ashkin et al., 1987). From these early experiments it was patent that employing visible light lasers (514.5 nm from an Ar-ion source) for trapping was much more damaging than infrared wavelengths (1.060 nm Nd-YAG laser) under similar circumstances. Thus, employing long wavelength (near infrared, NIR, ∼700-1200 nm) lasers to generate the optical traps was concluded to be less biologically interfering based on these results. The reason beneath this phenomenon will be introduced in the following sections (see sections “Mechanisms of Action of LS” and “Mechanisms of Damage in OT” below). For additional information on the history, fundamentals and applications of LS and OT the book *Laser manipulation of cells and tissues* (*Methods in Cell Biology* series) is highly recommended for the interested reader (Berns and Greulich, 2007). In the following sections the use of LS and OT for the case of genetic material manipulation will be presented. Given that, historically, LS were developed first, the first section will deal with this optical tool. Then, applications of OT for the same purpose will be introduced. Following this, another section will present experiments and results obtained with the combined action of LS and OT in Genetics and Cell Biology.

## Laser Scissors for Genetic Material Manipulation

In Cell Biology the possibility to selectively destroy or alter cellular components in a controlled way is very desirable. This is precisely what LS offer, with the additional advantages of very high spatial and temporal control of the ablative event and sterile conditions, as the laser light cannot be “contaminated,” biologically or otherwise. The different mechanisms taking place during the laser ablation will be briefly discussed before presenting relevant applications of LS for genetic material manipulation.

### Mechanisms of Action of LS

LS rest upon the phenomenon of laser microablation. It is important to, at least, have a general idea of the different mechanisms potentially leading to laser ablation. This will prepare the researcher to understand the advantages and drawbacks of the technique, and to better judge which irradiation parameters fit his or her necessities for a particular experiment. Photodamage can be classified in different ways. Here we will divide photodamage attending to three particular, although interconnected, processes: photochemistry, plasma generation and photothermal (photomechanical) (Quinto-Su and Venugopalan, 2007). Each one will be generally introduced in the following.

Photochemistry is chemistry taking place after one or more photons have been absorbed (Turro et al., 2010; Stockert and Blázquez-Castro, 2017). Molecules absorb a photon in the ground state and energize to one of several excited electronic states (the final state depends on the photon energy and relaxing mechanisms at work). The details are extensive and will not be treated here. Excited molecules are more reactive than ground state molecules, so they have a tendency to engage in chemical reactions usually not observed without photoexcitation. For example, DNA bases absorb photons with wavelengths below 300 nm. An UV microirradiation at those wavelengths can lead to crosslinking between bases, breaking of base-phosphate bonds (leading to abasic spots), or phosphate-sugar bonds (leading to single or double strand breaks). These events induce photochemical ablation within the irradiated volume after absorption of one photon per molecule. Efficient LS must provide very restricted (∼μm) interaction volumes if the damaged spot is to reveal how cells react to the insult. Therefore, use of UV beams is undesirable as many biological molecules absorb efficiently in this spectral range. At the cost of losing some tighter focusing, it is adequate to move to the visible and near-infrared (NIR) to better “constrain” the interaction volume. An issue here is that few biomolecules absorb in the visible, much less in the NIR. One alternative is to “dye” the sample with some chromophore molecule that will absorb photons at those wavelengths. This has been mentioned above in connection to the use of methylene blue or acridine orange, for example, to sensitize subcellular structures (e.g., chromosomes) to laser light (see section “HISTORICAL BACKGROUND”). However, this strategy introduces an artificial compound into the cell, which can alter its responses to the microirradiation.

A second alternative is to increase the photon flux across the interaction volume to achieve non-linear (or multiphoton) absorption and photochemistry (Quinto-Su and Venugopalan, 2007; Turro et al., 2010; Stockert and Blázquez-Castro, 2017). This is usually done by employing pulsed lasers with very short light emission, from nanosecond to femtosecond. During these very short pulses, photons traverse the focus volume at such high density that molecules absorb more than one photon at a time. This is non-linear photochemistry. For example, excited levels that would be populated through UV excitation can be produced with two, three or more visible/NIR photons, whose combined energy equals that of UV photons. Non-linear absorption only occurs within the focus volume, and as such, submicrometric microirradiation dimensions can be attained. This is one of the most important reasons why pulsed lasers are employed for LS, as they provide very high spatial and photochemical control of the irradiated volume.

Plasma generation (medium breakdown) can be considered a consequence of intense non-linear photochemistry when irradiance levels are larger than certain thresholds (Quinto-Su and Venugopalan, 2007). The phenomenon is complex, so only a general description is provided here. When enough photons are available under high-irradiance conditions, above 10^8^–10^9^ Wcm^–2^ in the optical range, the medium can simultaneously absorb a number of them, pumping molecules above their ionization potential. In consequence, free electrons appear in the medium which themselves are capable of absorbing more photons, initiating an avalanche ionization process (Noack and Vogel, 1999). Plasma generation commonly leads to high temperatures, elevated pressures and subsequent reactive chemistry and mechanical wave emission. However, if the laser plasma is excited and contained within a very small volume, the violent consequences of its generation affect only the immediate surroundings. This is a very adequate procedure to drive microablation in biological structures. Nowadays, most LS employed make use of plasma generation to achieve controlled ablation in biological experiments. It is noteworthy to mention that the mechanisms of plasma generation change when the laser pulses move down in temporal scale from nanosecond to femtosecond (Vogel et al., 2005; Linz et al., 2015). This is because of different, competing excitation processes overtaking one the others as irradiances increase with the shorter pulses. Whether avalanche ionization is the main ionization mechanisms for ns pulses, it is practically irrelevant for fs pulse ionization, favored by field-tunneling (Quinto-Su and Venugopalan, 2007; Vasa and Mathur, 2016; Linz et al., 2016). Partly because of this type of ionization mechanism, fs lasers allow for better control of the generated plasma, capable of producing very low-density plasmas of nanometric dimensions (few hundred nm) (Vogel et al., 2005, 2008). This is very advantageous for studies where submicrometric structure lesions are desirable for the study. The interested reader is directed to the cited bibliography for further information on these topics.

Finally, photothermal effects are a consequence of photoexcitation, as some fraction of the optical energy always appears as heat in the irradiated medium (Vogel and Venugopalan, 2003; Vogel et al., 2005). This heat can directly affect molecules and cellular structures, favoring molecular denaturation for example, or can produce mechanical waves due to the sudden expansion of the heated volume, disrupting nearby or distant structures (Venugopalan et al., 2002). As researchers employing LS usually try to confine as much as possible the ablation effect within a small volume, photothermal/photomechanical action is seen as a nuisance in most experiments. Therefore, it is rare to rely on the photothermal effect to achieve precise laser ablation of the cellular structures discussed in this review. Nevertheless, it is important to remark that the photothermal (and associated photomechanical) effect is always present, to a larger or smaller extent, in all experiments using LS.

After briefly summarizing the main mechanisms at action in the LS, now different applications of this versatile tool will be presented in relation to the manipulation and alteration of genetic material at different levels of organization: mainly chromosomes and chromatin, nuclear structures, and DNA in the DNA damage response (DDR).

### Chromosome Cutting and Microdissection

Condensed chromosomes have typical dimensions of a few microns long by less than one micron wide. Therefore, LS present as very suitable tools to cut and dissect these structures, given their sharp ablating limit down to 0.4–0.5 μm (Heisterkamp et al., 2007). On the other hand, if necessary, it is possible to widen the laser focus and/or scan it across the sample to achieve ablation of larger structures. For example, experiments dealing with these two limiting cases were published almost at the same time already in 1969. In one case, Berns et al. reported the possibility to selectively damage mammalian chromosomes down to an estimated lesion size of 0.6–0.8 μm with their early LS system (Berns et al., 1969a,b). In parallel, McKinnell et al. were able to eliminate whole maternal chromosomes, several microns in extension, in an embryonic amphibian model employing a pulsed ruby laser (McKinnell et al., 1969). The treated eggs developed into haploid embryos for a time, starting their development as diploid at fertilization before the laser treatment.

At first, these pioneering works had to depend on some “dyeing” procedure or fortuitous absorbing structure to enact the microablation. Berns et al. employed acridine orange or ethidium bromide to increase chromosomal laser absorption. McKinnell and collaborators relied on the “blackened” structure (as they described it) naturally formed by the second meiotic division spindle. Soon, new laser systems were developed, which offered better suited irradiation wavelengths and packed more power to work above the non-linear photochemistry threshold (Berns et al., 1976). With these systems, no artificial chromophores were now necessary to increase absorption and damage the chromosomes. More importantly, irradiated mitotic cells completed their cycle and the impact of chromosome microirradiation could be assessed, for example, by observing their capacity to enter additional cell cycles or overall cell survival. Biochemical tests showed that, depending on the irradiation conditions, the nucleic acid or the protein component of chromatin was preferentially damaged by the LS. Under more intense exposures, both chromatin components were damaged. Indeed, it was possible to completely detach a chromosome region, leaving a physically independent chromosome fragment which could be further isolated for analysis (see section “COMBINED LS AND OT FOR GENETIC MATERIAL MANIPULATION” below).

**FIGURE 2:**
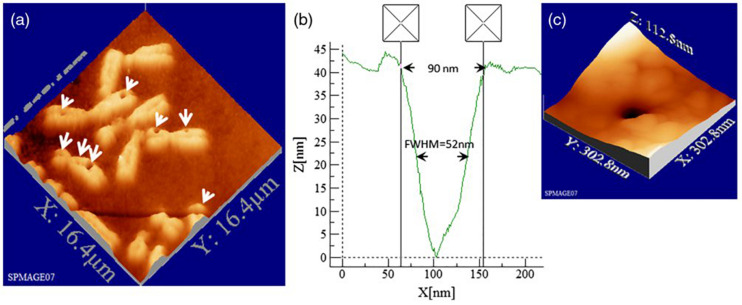
Topographic AFM (atomic force microscope) images showing cutting and drilling effects of the laser after processing on chromosomes. In subpanel **(a)** the arrows show laser produced holes with different diameter size and cut incision. In subpanels **(b)** and **(c)** the measured profile from a hole with a diameter of 90 nm (FWHM of 52 nm) is displayed in one dimension **(b)** and three dimension **(c)** plots. Reproduced with permission from Uchugonova et al. (2012).

Soon, the technique matured into the so called chromosome microdissection, with opened multiple opportunities for chromosomic analysis and establishment of genetic libraries from selected chromosomic fragments (Greulich, 1992; Schütze et al., 1997; Ronchi et al., 2012; Greulich, 2017). In microdissection, a particular chromosome from a chromosome sample (for example, a chromosomal spread) is selected for analysis under microscopic scrutiny. Surrounding chromosomes are first ablated with the LS to minimize interference. Then, the region of interest of the chromosome is isolated by carefully scanning the LS across the chromosome. Usually, once this is done, the rest of the chromosome is eliminated with the LS to avoid sample contamination. Finally, the microdissected fragment is retrieved by some means for analysis. A commonly employed technique is laser catapulting, which focuses the laser on the substrate from below the sample for ejection into some receiving container. Then, analysis of the isolated fragment can proceed by different techniques. It has been possible to microdissect and analyze human chromosomes (Monajembashi et al., 1986; Eckelt et al., 1989; Hadano et al., 1991; He et al., 1997), as well as map chromosomal regions linked to certain disorders, such as fragile X chromosome (Djabali et al., 1991) or muscular dystrophy (Upadhyaya et al., 1995). More recently, the adoption of femtosecond lasers for LS made it possible to ablate very small chromosome regions, initially down to 200 nm (König et al., 2001), and more recently to less than 100 nm (Uchugonova et al., 2012). An example of such precise cutting is shown in metaphasic chromosomes (Figure 2). Subpanel a shows an AFM plot of irradiated chromosomes. The white arrows indicate the points of laser cutting (holes). Some cuts are smaller than 100 nm (examples in subpanels b and c). This is advantageous because the less material is ablated to produce the cut, the less chromatin is lost for posterior analysis of the fragment.

**FIGURE 3:**
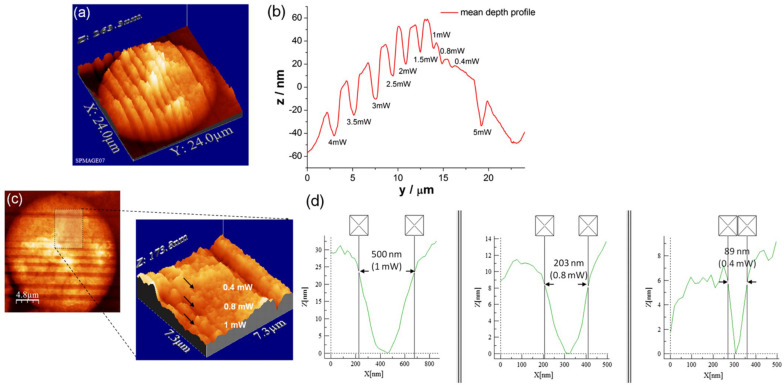
LS ablation streaks of interphasic chromatin. Subpanel **(a)**: 3-D AFM scans of line cuts in a cell nucleus realized with 12 fs laser pulses at mean powers (mW) of 4.0, 3.5, 3.0, 2.5, 2.0, 1.5, 1.0, 0.8, 0.4, and 5.0. Subpanel **(b)**: Depth profiles (nm) of the nuclear cuts displayed in **(a)**. The graph (mean profile line) indicates that a low laser power of 0.4 mWwas sufficient to induce ablation effects. Note the different spatial scale between plot axes. Subpanel **(c)**: Magnified AFM image with three incisions at 0.4, 0.8, and 1 mW. Subpanel **(d)**: Depth profiles of the three lowest intensity incisions (1, 0.8 and 0.4 mW) displayed in **(c)**. Analysis of individual line cuts of the cell nucleus shows that sub-100 nm lines can be created with 12 fs laser pulses. Note the different spatial scale between plot axes. Reproduced with permission from Uchugonova et al. (2012).

Similar precision can be obtained in the interphasic nucleus (Figure 3; Uchugonova et al., 2012). Very defined linear laser cuttings of different widths were obtained by scanning at different average laser powers (subpanels a and b). By carefully choosing the laser power (1 mW or less), extremely thin sections could be ablated in the interphasic chromatin (subpanel c), even below 100 nm (subpanel d).

Genetic material from other organisms has also been microdissected with LS. For example, insect chromosomes (Diptera) have been successfully ablated and analyzed. Chromosomes from *Drosophila melanogaster*, one of the most employed genetic animal models, were microdissected and, then, sequences were compared to data bases to check that the technique was robust for genetic analysis (Ponelies et al., 1989). Microdissection has proven successful in the establishment of correlations between the visible structures of the chromosomes and the DNA sequence contained therein. This facilitated the use of molecular markers for population genetics and cytotaxonomy in the disease-vector blackfly (*Simulium thyolense*) (Post et al., 2006), with relevant implications in disease control strategies.

**FIGURE 4:**
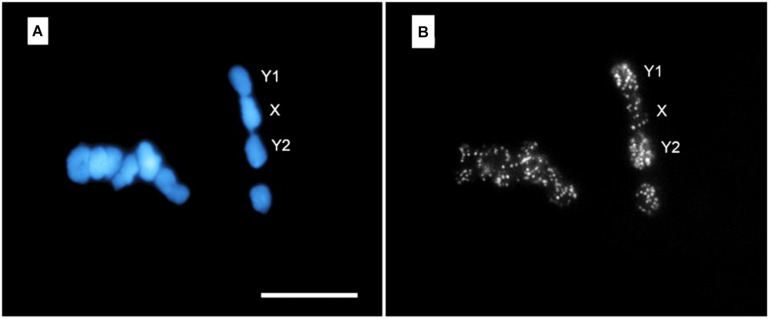
FISH with DOP-PCR (degenerate oligonucleotide primed-PCR) probe on meiotic chromosomes of *H. japonicus*. Subpanel **(A)** shows DAPI-stained chromosomes at meiotic metaphase I stage. Note the micrometric cuts (darker stripes) executed in each chromosome. In subpanel **(B)** the result of FISH with DOP-PCR probes (bright spots) is shown. The Y1-X-Y2 trivalent formation is indicated. Bar = 10 μm. Reproduced with permission from Yakovin et al. (2014).

Microdissection has been particularly fruitful in the cytogenetic analysis of plants (Day et al., 2007; Hobza and Vyskot, 2007). This can have profound consequences, as genetic selection and engineering of edible plants is at the base of the food production chain for the whole world population. Microdissection by LS has been successfully applied to wheat (Zan-Min et al., 2004), barley (Fukui et al., 1992), rice (Fukui et al., 1992; Liu et al., 2004; Wang et al., 2004), and orchid plants (Balestrini et al., 2018), all of them with very relevant economic impact. It has also been applied to study the complex sexual chromosomes arrangement of certain plant species (Matsunaga et al., 1999; Yakovin et al., 2014). Additionally, it is worth noting that LS microdissection has been combined with established genetic analysis techniques, such as polymerase chain reaction (PCR) (Hadano et al., 1991; Meimberg et al., 2003; Zan-Min et al., 2004; Post et al., 2006) and fluorescence *in situ* hybridization (FISH) (Lengauer et al., 1991; Métézeau et al., 1995; Rajcan-Separovic et al., 1995; Hobza and Vyskot, 2007). Shown in Figure 4 is an example of LS microdissection of *H. japonicus* sexual chromosome followed by identification by means of FISH probes (Yakovin et al., 2014). Subpanel a shows the overall appearance of the microdissected chromosomes in fluorescence microscopy. In subpanel b, a large number of positive FISH probes hybridizations are displayed, as testified by the number of bright spots in each chromosome fragment.

These techniques offer very high resolution and control over the genetic material to be sampled. However, this comes at the price of having to implement optical platforms (microscope, lasers, optical components, etc.) which need some period of training and use before their potential for genetic analysis becomes patent. Also, most of the examples in this review, discussed here and later, of genetic manipulation by LS and OT require longer manipulation times than other techniques, because it is necessary to individually choose and manipulate each sample of interest. Nevertheless, progress in automated platforms capable of speeding up these steps is on its way (see section “PERSPECTIVES” below).

### Nuclear Structures Microirradiation

Another important use of LS has been the microirradiation of particular nuclear structures, which can be seen as an alteration of the chromatin and chromatin-protein complexes (e.g., the nucleolus) in order to perturb a cell (Berns, 1978, 2007a; Greulich, 2017). This was a very productive research line for Berns and collaborators in the 1970s (Berns and Rounds, 1969; Berns et al., 1970, 1971, 1972; Berns and Floyd, 1971; Ohnuki et al., 1972; Rattner and Berns, 1974). The nucleoli offer a small (∼1 μm) but clearly discernible target for laser microirradiation. Being the spot(s) where DNA transcription related to ribosome assembling and protein translation takes place, its more or less intense disruption provides a condition capable of visibly altering a cell’s behavior, like mitotic blockade, cellular senescence or death. Berns and collaborators were capable of establishing cell sub-lines which stably lacked some nucleoli from microirradiated single cell progenitors (Berns, 1974; Berns et al., 1979; Liang and Berns, 1983a,b). A very interesting outcome of nucleolus microirradiation was reported in 1989 (Hu et al., 1989). Laser disruption of nucleoli in PTK2 cells led to *de novo* assembly of a certain number of “subsidiary” micro-nucleoli between 12 and 24h after treatment. This was hypothesized as a cellular rescue response by which formerly repressed nucleolar organizing regions were activated after disruption of the main nucleolus. Later, nucleolar disruption with 100% short term (24h) cell survival was achieved under subtler microirradiation conditions, relying upon a bi-photonic process (Berns et al., 2000). This should be a less damaging photo-treatment in comparison to plasma generation, which has been the classical ablative process for most LS experiments. However, modern femtosecond lasers can offer highly contained low-density plasmas, which should also be considered a relatively gentle treatment (see section “Mechanisms of Action of LS” above). New technologies, in combination with LS, pave the way for less aggressive and more delimited treatments. This is the case, for example, of digital holographic microscopy, which has been employed with LS to ablate a nucleolus under more environmentally friendly conditions for the cells, adapting in real time to microscopic changing conditions, like cell movement, to maintain a stable irradiation spot (Yu et al., 2009).

Aside from the very prolific nucleolar and condensed chromosomes irradiation experiments, LS have been put to use to determine if interphasic chromosomes occupy particular regions in the nucleus or, on the contrary, they are intermingled across the nuclear volume (Cremer et al., 1982). To answer this question an UV 257 nm laser was focused to relatively small spots (1 μm) of the cell nucleus. Afterward, genetic damage repair was measured for each cell at different times. It was observed that only one or very few chromosomes were damaged by each treatment. This gave support to the idea that each chromosome occupies a particular region in the nucleus and are kept relatively isolated one from another. When microirradiations took place at the nuclear edge, close to the nuclear envelope, the distal regions of chromosomes and the telomeres were significantly more damaged than the centriolar/central parts. This provides evidence that chromosome docking to the nuclear envelope takes place at the distal chromosome regions. This experiment is an elegant example of how an alteration of the genetic material (interphasic chromatin in this case) by LS damage induction can serve as a “pulse tracer” approach for analysis of structural features. A more recent publication reported a similar strategy to study mitotic damage checkpoints by microirradiating the chromosome tips at particular moments during mitosis (Baker et al., 2010). Chromosome tips (peri-telomeric regions) were laser-exposed (532 nm, 12 ps) when target cells were in anaphase. Equivalent irradiations were also done on non-distal chromosome regions, the nuclear volume close to but not directly overlapping chromosome tips, or the cytoplasm. It was confirmed that only when laser damage was done to peri-telomeric spots a rescue mechanism was actuated that immediately blocked mitosis before cytokinesis, or greatly interfered with it. Moreover, the blocking response strength was dose-dependent on the number of chromosomes affected per irradiated cell. This was proof of a specific mechanism monitoring telomere kinetics and integrity during ana-telophase, capable of enacting a blocking response if an aberrant situation is detected to avoid a faulty mitotic exit.

### DNA Damage Response and Nuclear Perturbation

Another very successful area of application of LS has been the assessment of the DNA damage response (DDR). The DDR is a generic term that encompasses several overlapping cellular responses to different kinds of DNA and chromatin damage (Heijink et al., 2013; Bauer et al., 2015; Ferrari and Gentili, 2016). As there are so many different kinds of insults capable of damaging the genetic material (chemicals, reactive oxygen species – ROS-, mechanical perturbations, heat shock, ionizing radiation, etc.), it is acknowledge that the topic is quite broad. Here, some relevant experiments and results in relation to the use of LS for the study of DDR will be presented. But the interested reader is encouraged to consult the bibliography for further information in the field. In the context of DDR and nuclear perturbation LS acts at a less intensive level, damaging the target structure but not dissecting it. The type(s) of damage dealt depends on the microirradiation conditions (wavelength, irradiance, dwell time, etc.). The cellular recovery processes set in motion by the LS can be studied on a cell-by-cell basis or over a sample of many cells under more automatized conditions.

A very important asset of LS in the study of the DDR is the wide range of different biological lesions induced depending on the irradiation parameters. By tuning the laser wavelength, pulse duration, dwell time and scanning frequency, pulse energy and power, irradiance, or presence/absence of photosensitizing molecules different damage patterns and lesions are observed (Kong et al., 2009). Table 1 shows selected examples of different kinds of genetic lesions (oxidized DNA bases, base crosslinking, single strand breaks (SSB), or double strand breaks (DSB) induced by different LS setups. This is meant only as an example of the flexibility of the system. More information can be found in the bibliography. To deal with this plethora of genetic lesions, the cell activates a series of different DDR proteins and signaling pathways (Bauer et al., 2015; Ferrari and Gentili, 2016). Thus, LS offer a very versatile experimental approach to study this highly relevant field involved in such areas as cell mutation, aging, cancer, radiobiology, development, or regeneration (Gomez-Godinez et al., 2007; Grigaravičius et al., 2009; Drexler and Ruiz-Gómez, 2015).

**TABLE 1:** Genetic lesion types induced by LS.

Genetic lesion	Cell line	LS conditions	Remarks	References
oxidized DNA bases	HeLa	337 nm, pulsed (4 ns), ∼315 nm-wide scanning line (BrdU sometimes employed)	8-oxoG	Kong et al., 2009
base crosslinking	HeLa	337 nm, pulsed (4 ns), ∼315 nm-wide scanning line (BrdU sometimes employed) 532 nm (12 ps), ∼465 nm-wide scanning line, NIR fs 800 nm (200 fs), ∼700 nm-wide scanning line	Detection of 6-4 pyrimidine-pyrimidone photoproducts (6-4PP) and cyclobutane photodimers	Kong et al., 2009
	HeLa	515 nm, pulsed (81 fs), 4.6 μm-long line within nucleus	Induction of cyclobutane photodimers (CPD)	Schmalz et al., 2018
strand single breaks (SSB)	HeLa	337 nm, pulsed (4 ns), ∼315 nm-wide scanning line (BrdU sometimes employed) 532 nm (12 ps), ∼465 nm-wide scanning line 532 nm (6 ns), ∼500 nm-wide scanning line, NIR fs 800 nm (200 fs), ∼700 nm-wide scanning line	Induction of PARP-1, XRCC1 and FEN1	Kong et al., 2009
double strand breaks (DSB)	HeLa and human fibroblasts	532 nm, pulsed (4-6 ns), scanning 5 μm line, 2 min/cell	Role of cohesins in DDR	Kim et al., 2002
	PTK1 and CFPAC-1	800 nm, pulsed (200 fs), 20 ms pulse/cell	Detection of DSB by pH2AX, recruitment of Nbs1 and Rad50	Gomez-Godinez et al., 2007
	HeLa	337 nm, pulsed (4 ns), ∼315 nm-wide scanning line (BrdU sometimes employed) 532 nm (12 ps), ∼465 nm-wide scanning line, NIR fs 800 nm (200 fs), ∼700 nm-wide scanning line	Activation of PARP-1, Ku70 and pH2AX	Kong et al., 2009
	HeLa	1035 nm, pulsed (81 fs), 4.6 μm-long line within nucleus	Activation of pH2AX	Schmalz et al., 2018

*BrdU, 5-bromo-2′-deoxyuridine; 8-oxoG, 8-Oxoguanine; PARP-1, Poly(ADP-ribose) polymerase-1; XRCC1, X-ray repair cross-complementing protein 1; FEN1, Flap endonuclease 1; PTK1, Rat kangaroo kidney epithelial cells; CFPAC-1, human cystic fibrosis pancreatic adenocarcinoma cells; pH2AX, phosphorylated-H2A histone family member X; Nbs1, Nibrin protein; Rad50, DNA repair protein Rad50; Ku70, Ku70 protein.*

**FIGURE 5:**
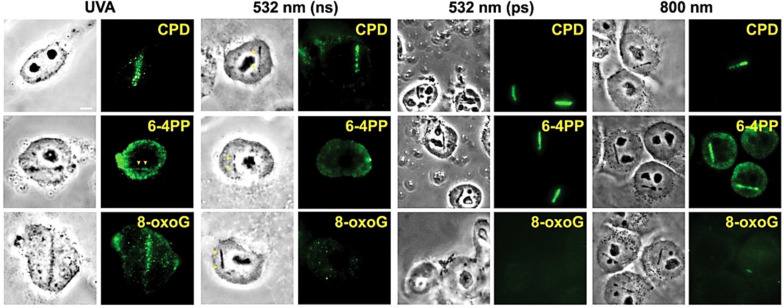
Induction of different types of DNA damage by UVA, ns and ps green, and NIR lasers. At 3–5 min after damage induction by the different lasers indicated at the top, cells were fixed and stained with antibodies specific for CPD, 6-4PP and 8-oxoG. Corresponding bright field phase contrast images are also shown. Scale bar = 5 μm. Reproduced with permission from Kong et al. (2009).

As mentioned, using different lasers and/or irradiation parameters different lesions and mechanisms are induced (see section “Mechanisms of Action of LS” above). For example, UV lasers can directly produce DNA damage (e.g., photodimers) due to the direct absorption of photons with wavelengths below 300 nm. Long-range UV (300–400 nm) and visible (400–700 nm) lasers can produced direct and indirect (oxidative) damage when light absorbing compounds are present at the time of irradiation. A typical example of this approach is to sensitize DNA with the base analog 5-bromo-2′-deoxyuridine (BrdU, see Table 1), a compound absorbing UV photons and inducing DNA alterations (base modifications, strand breaks, etc.). Pulsed lasers can produce plasmas that also induce a series of lesions in the nearby genetic material. Kong et al. (2009) compared several laser systems in order to check similarities and differences as to the type of genetic lesion induced. They compared a pulsed ns 337 nm laser (with or without BrdU), a continuous wave (cw) 405 nm laser (with or without BrdU), two 532 nm pulsed (ns and ps) lasers, and a pulsed fs 800 nm laser (see Table 1 for more details). They found that UVA laser was particularly effective in producing oxidized or photodimerized bases, while 532 nm and 800 nm lasers were more prone to induce DNA strand breaks. Some examples of the different genetic lesions are displayed in Figure 5.

Another interesting example of the potential of LS for DDR study has been recently reported. In this case the authors employed lasers with three different wavelengths (515, 775, or 1035 nm) but exactly the same pulse length (80–81 fs) (Schmalz et al., 2018). Under conditions of similar average power, different alterations of the chromatin were observed in the irradiated cells. For example, irradiation at 515 nm gave rise to cyclobutane pyrimidine dimers, while light of 1035 nm greatly increased the levels of strand breaks. This is experimental proof that selective genetic lesions can be induced under suitable microirradiation parameters, with obvious implications for studying the cell reaction to those selective lesions. Moreover, as each irradiation scan lasted 4.68 s, nothing in principle precludes the induction of two or more types of lesions in the region of interest in a fast sequential irradiation with different wavelengths. Or the analysis of the cell reaction to different lesions in different zones of the same nucleus.

**FIGURE 6:**
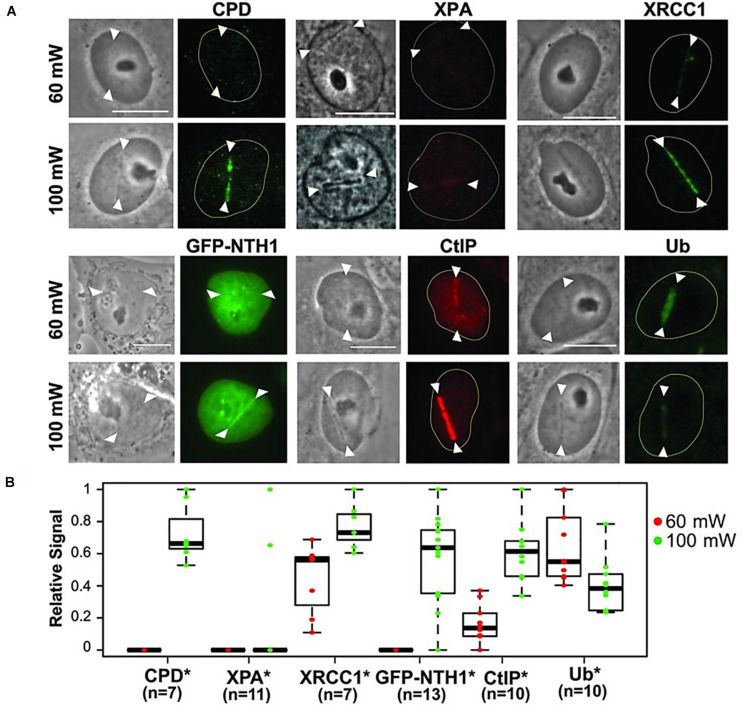
**(A)** Characterization of damage induced by low and high input power laser microirradiation. Interphase PtK2 cells were irradiated at 60 and 100 mW input powers, corresponding to ≈2.1 × 10^11^ W/cm^2^ and ≈3.49 × 10^11^ W/cm^2^ peak irradiances, respectively. Cells were fixed and stained with antibody specific for CPD (*N* = 7 each for 60 and 100 mW; cells were fixed at 10 min p.i.), XPA (*N* = 11 each; cells were fixed at 3 min p.i.), and the SSB repair protein XRCC1 (*N* = 7 each; cells were fixed at 5 min p.i.). PtK2 cells expressing GFP-NTH1 were also irradiated at 60 and 100 mW input powers and were followed for 1 min (*N* = 13 each), Scale bar = 10 μm. Immunofluorescent detection of the DSB end-resection factor CtIP and ubiquitin (Ub) at 60 and 100 mW damage sites at 30 min p.i. (*N* = 10 each) was also performed. Representative images (including the live cell images of GFP-NTH1, indicative of base excision repair) are shown for the factors indicated at the top. **(B)** Quantitative fluorescent intensity measurements of the damage-site recruitment were done and were displayed relative to the highest signal observed within in each group underneath. Asterisks confirm the significant *P*-values (< 0.05) for the differences of the factor recruitment between 60 and 100 mW. Reproduced with permission from Saquilabon Cruz et al. (2016).

A further illustrative example is shown in Figure 6. The researchers studied the genetic damage profile after microirradiating under the same conditions (laser, pulse length, wavelength), but just changing the laser power between high dose (100 mW, 3.49 × 10^11^ W cm^–2^) and low dose (60 mW, 2.10 × 10^11^ W cm^–2^) (Figure 6A; Saquilabon Cruz et al., 2016). It can be seen that SSB with a certain biochemical pattern (XRCC1 and Ub activation) are induced at a low dose (Figure 6B). In contrast, a high dose led to a more complex SSB profile (XRCC1, GFP-NTH1 (chimeric GFP-Endonuclease III-like protein 1), CtIP and Ub) as well as the appearance of cyclobutane photodimers (CPD).

These experiments show the relevance that different irradiation parameters have on the damage mechanism(s) created by the LS (see section “Mechanisms of Action of LS” above). For example, Kong et al. (2009) made a much elaborated discussion on the possible photo-processes (single-photon, multi-photon, photothermal, plasma generation) taking place at irradiation spots, and correlated them to the different genetic lesions observed. Other authors have also made pertinent microirradiation parameters-genetic lesions correlations (Gomez-Godinez et al., 2010; Zielinska et al., 2011; Ferrando-May et al., 2013; Gassman and Wilson, 2015). From these studies it can be concluded that certain experimental parameters favor the production of one type of lesion over others. This is an advantage as compared to other classical lesion-inducing approaches, like ionizing radiation or photodynamic treatments, in which less control is possible over the induced chemical reactions. Also, there is the possibility to study responses to the treatment at the single cell level and of particular genetic structures in the cell (e.g., a concrete chromosome region of a single chromosome) due to the microirradiation capabilities of the laser systems. The approach, however, is not without drawbacks. Cell-by-cell microirradiation makes the experimental procedure slow and comparing large cell populations is lengthy. Additionally, there is still low control over the types of lesions produced depending on the damaging mechanism exploited to carry out the experiments. For example, better lesion control is obtained with UV-excitation of BrdU than with microplasma induction (see Table 1). Anyhow, a microplasma mimics more realistically the events taking place under ionizing radiation exposure, in particular heavy particles like neutrons, alpha particles or ions, which can be considered an advantage depending on the research objectives (Botchway et al., 2010).

**FIGURE 7:**
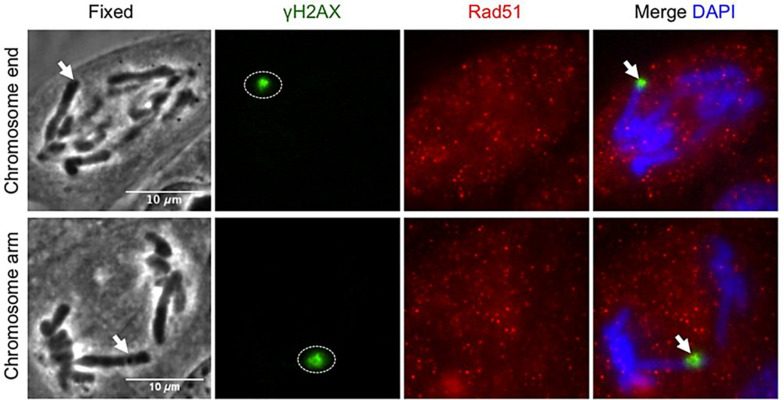
Homologous repair protein Rad51 does not get recruited to anaphase DNA breaks. Postfixation performed 5 min after laser irradiation to a single chromosome end and chromosome arm. Repair proteins are detected with anti-γH2AX (green), anti-Rad51 (red), and co-stained with DAPI (blue). Arrow points to microirradiated chromosome site. γH2AX: phosphorylated-H2AX; Rad50: DNA repair protein Rad50. Scale bar = 10 μm. Reproduced with permission from Alcaraz Silva et al. (2013).

We would like to make a final remark on some experiments dealing with microirradiation of the telomeres. The telomeres are the most distal regions of the chromosomes and have many important roles in regards to different cellular aspects. They “keep track” of the number of division rounds a cell has undergone, have a fundamental role in mitotic coordination, and are hot spots for chromatin damage, cellular senescence, genomic instability and cancer development. In consequence, the possibility to employ LS to induce controlled damage to one or more particular telomeres is a very powerful tool to study cell behavior in the above mentioned areas. The work by Baker and collaborators has been already discussed above in regards to this (see section “Nuclear Structures Microirradiation” above) (Baker et al., 2010). In connection to DDR, it has been shown that making use of LS to microirradiate telomeres engages the DDR (due to induction of SSB and DSB), and interferes with mitotic cycle progression of the affected cell (Alcaraz Silva et al., 2013, 2014; Figure 7). Notably, in some instances, the telomeric repair mechanism is defective and damaged telomeres progress into G1 (Alcaraz Silva et al., 2013). Even more concerning, these unrepaired telomeres end up producing a significant increase of micronuclei in the affected cell (Alcaraz Silva et al., 2014). This has very grave consequences for the genomic stability of the cell, with rising chances of it progressing toward a tumoral type. More on the potential applications of this research line in regards to the study of genomic instability will be provided in the section “PERSPECTIVES” below.

In the following relevant approaches to genetic material manipulation using optical tweezers (OT) will be presented.

## Optical Tweezers for Genetic Material Manipulation

There was a considerable lag between the moment OT were proposed and first proved (Ashkin, 1970) and the first OT application to biological samples (Ashkin and Dziedzic, 1987; Ashkin et al., 1987). However, once proof of principle was provided, the technique gained popularity quickly and many laboratories started research projects employing OT. This section will be structured as follows. First, the principles of action of OT will be very briefly introduced. Then, important information on the potential sources of damage in OT will be presented, along with some strategies to be implemented in order to minimize this OT-derived damage. Lastly, some examples of genetic material manipulation and analysis with OT will be provided.

The mechanism of action of OT depends, in part on the ratio between the size of the object to be trapped and the wavelength of the light employed in the OT, and in part on the difference between the refractive indexes of the object to be trapped and the surrounding medium (Berns and Greulich, 2007; Gao et al., 2017; Greulich, 2017; Dhakal and Lakshminarayanan, 2018). OT trapping can be generally divided in two regimes attending to the size ratio of the object to the light wavelength: the ray optics (geometrical regime) when the object is much larger than the light wavelength, and the induced-dipole (Rayleigh regime) when the object is much smaller than the wavelength (Bowman and Padgett, 2013; Bradac, 2018). In the ray optics regime the deflection of light beams, as they traverse the trapped particle, results in a net force that moves said particle toward the region with the highest irradiance (optical focus). This is because photons carry not only energy but also momentum. Therefore, when they change their propagation direction they exert a momentum on the object that forced them to such change. On the other hand, when the trapped particle is smaller than the wavelength of light, the ray optics regime is no longer valid and one has to rely upon the induced-dipole regime. The trapped particle, partially or completely embedded in the trapping light beam, develops transient electric dipoles as a result of the very high electric field associated with the focused trapping light. Due to the dielectrophoretic effect these induced dipoles force the particle to migrate to the volume of highest electric field, in this case the optical focus. It is seen that both regimes result in the particle being transported to the optical focus of the trap. In general, these two approximations are valid for particles whose refractive index is higher than that of the surrounding medium. If the refractive index is lower, then the opposite reaction takes place and the particle would move away from the OT. Genetic material (DNA, chromatin) is commonly trapped by OT in aqueous media, therefore its refractive index is higher than that of water and trapping occurs. The fundamentals and variants of OT is a very wide topic, too extensive to be discussed here. We refer the interested reader to the cited bibliography for additional information. We will now introduce in more detail another topic which is more relevant for this review: the sources of damage in OT and some ways to reduce their impact upon the trapped sample.

### Mechanisms of Damage in OT

In contrast to LS, where damage is purposefully sought to alter or manipulate the biological sample, damage is generally avoided when using OT. This is because OT should trap or displace the trapped object with minimal interaction over it. Of course, the light must interact with the object to trap it, at the very least to impart momentum or induce dipoles. Given the very high irradiances necessary to interact with the object in these ways (10^3^ W cm^–2^ or higher), even a very small light absorption can be enough to initiate photochemistry or other undesired processes. As some level of unwanted interaction will occur, it is necessary to know the general types of damage that the OT can produce and the measures available to reduce their impact on the sample. Note that the damage mechanisms to be briefly introduced below are basically the same that were discussed at more length for LS (see section “Mechanisms of Action of LS” above). Thus, the reader can also consult that section for more information. Additionally, a review on this particular topic of damage mechanisms in OT has been recently published, providing detailed information (Blázquez-Castro, 2019).

Two are the main types of deleterious processes that will occur in OT: photochemistry and photothermal effects (Berns, 2007b; Norregaard et al., 2014). Photochemistry is the most obvious source of damage in OT. As mentioned, very high photon fluxes cross the trapped object per unit time in OT. Therefore, both linear and non-linear photochemistry will be taking place in a more or less intense way. Photochemistry starts with photon absorption. In consequence, reducing any absorption event in the sample will limit photochemistry. Control over the absorption can be achieved by judiciously selecting the laser wavelength to avoid anticipated one- or multiple-photon absorption. This is the reason why practically all OT systems at present use NIR emission, to avoid excitation in the visible and the UV. Reducing the irradiance drastically decreases non-linear photochemistry. This is achieved, whenever possible, by reducing the laser power, the degree of light focusing, and/or using a cw source as opposed to pulsed ones. From these measures one can conclude that the desirable parameters for non-disrupting OT are the opposite as those required for LS.

A call of attention is necessary at this point. As indicated, the majority of laser sources for OT emit in the NIR (700–1300 nm). Indeed, these sources have shown the best performance for this task. However, some molecular oxygen absorption bands do occur in the NIR (Blázquez-Castro, 2017). The most relevant are at 760–765 nm, 1060–1070 nm, and 1240–1270 nm. Many OT systems currently in use have a Nd:YAG laser as the light source, which emits at 1064 nm. This falls within one of the oxygen excitation bands. Less common, but exploited by a few groups, some OT have been implemented with light in the 740–820 nm range. Indeed, some damage induced around 760 nm of unexplained source was reported in the past in several publications (Vorobjev et al., 1993; Liang et al., 1996; Blázquez-Castro, 2019 and references therein). Most likely this damage was the result of singlet molecular oxygen (^1^O_2_) excitation at ∼760 nm. This wavelength has been recently employed to provoke severe damage and cell death by microirradiation, and systematically proved to be due to direct optical generation of ^1^O_2_ (Bregnhøj et al., 2015; Blázquez-Castro et al., 2020). Therefore, a cautionary warning call is made here for researchers, in order to avoid those wavelengths in OT to reduce oxidative damage to the manipulated biological samples. Apart from avoiding the molecular oxygen absorption bands, adding antioxidants or ^1^O_2_ quenchers can greatly help to reduce the impact of molecular oxygen activation by OT (these strategies are elaborated at length in Blázquez-Castro, 2019).

The other relevant source of damage in OT is the production of heat due to laser light absorption: the photothermal effect (Berns, 2007b; Blázquez-Castro, 2019). Heat production starts with photon absorption. Then, all or part of the photon’s energy will degrade into molecular vibrations and random movement, increasing the temperature. As with photochemistry, the best strategy to reduce heat production is to minimize light absorption. It is important to consider that most experiments using OT to manipulate biological samples (including genetic materials) take place in aqueous environments. Water features some absorption bands in the NIR that should be avoided in OT setups, for example, at 970–980 nm (Haro-González et al., 2013). The much employed 1064 nm laser line provides a mild photothermal effect, also due to water absorption. Theoretical analysis and experimental measurements show that a temperature increase of ∼10°C per watt of optical power is expected for this wavelength (Berns, 2007b; Norregaard et al., 2014). Commonly, between 100 and 500 mW are employed in the OT, so temperature should not rise above a couple of degrees at the focus. Nevertheless, it is important to consider that huge spatial-thermal *gradients* (> 10^6^ K m^–1^) can be established and maintained by OT, even for a small temperature difference. This can have undesired biological effects (Blázquez-Castro, 2019). Apart of correct OT wavelength selection, active (e.g., micro flows) and passive (e.g., heat-conducting structures) measures can be taken to increase heat dissipation in the sample.

Finally, other less obvious sources of biological perturbation, like mechanical, acoustical or vibrational, can occur under the right circumstances in an OT setup (Blázquez-Castro, 2019). The researcher should at least have these in mind, in order to correctly interpret unexpected experimental results. In the following, selected examples of genetic material manipulation with OT will be presented.

### Genetic Material Manipulation

Optical tweezers are successful for the manipulation of mitotic chromosomes and the mitotic apparatus (spindle, microtubules, cytoskeletal network, etc.). As a very recent review has been published dealing extensively with these matters (see Berns, 2020 in this special issue), we will deal here with complementary uses in other systems (e.g., bacteria or interphasic nuclei) in which genetic material has also been manipulated by OT. For biomechanically oriented results and studies of the DNA molecule itself or interacting with certain other biomolecules under *in vitro* conditions, the review by Heller and collaborators is recommended (Heller et al., 2014).

Large, internal cellular structures with sufficient refractive index difference as compared to their surroundings can potentially be trapped by OT and moved, if a relative displacement between the trapping laser and the sample can be established (Norregaard et al., 2014). The nucleus in eukaryotic cells is the largest structure, and its dense composition and compactness increases its refractive index above that of the surrounding cytoplasm and other organelles. As such, the nucleus makes an interesting structure to be trapped and moved inside living cells by OT. This was indirectly achieved by Aufderheide et al. (1992) and collaborators by positioning the nuclei in living *Paramecium tetraurelia* protozoa with the help of a cw 1064 nm OT. To move the nuclei inside the cells it was necessary to focus the laser on the internal crystals commonly found in this species. Then, by moving these crystals until contact was establish with a nucleus, it was possible to push and drag the nucleus around. Moreover, no damage was reported in the organisms during or for some time after interrupting the optical manipulation (Aufderheide et al., 1993). Ketelaar et al. (2002) successfully applied the technique directly to nuclei in plant cells when they optically trapped nuclei in *Arabidopsis thaliana* root hair cells. In order to assess the role of actin in the root hair growth, the researchers trapped nuclei in certain cells and maintained their spatial position fixed with the OT, while the rest of the cell kept growing. They were able to measure the microscopic forces exerted and that actin played a key role for the coordinated apical growth of the cell.

The nucleus has also been indirectly trapped and internally moved in cells of the fission yeast *Schizosaccharomyces pombe* employing OT (Sacconi et al., 2005; Tolić-Nørrelykke et al., 2005). Previously, it has been shown that submicrometric (∼300 nm) lipid granules within the cells could be robustly trapped with OT (Tolić-Nørrelykke et al., 2004). Then, these optically trapped lipid granules were employed as an internal “paddle” to push on the nucleus and displace it (Figure 1A). With this methodology nuclei were moved within cells. Furthermore, by controlling the nuclear position at particular times during the cell cycle, the division plane establishing the daughter cells polarity after cell division could be externally manipulated with the OT. This nuclear trapping is a very interesting technique which can have relevant ramifications for cell signaling and control (see section “PERSPECTIVES” below for more on this).

Other organelles carrying genetic information have been successfully trapped with OT. For example, Aufderheide et al. (1992) mentioned in their paper on optical positioning of nuclei in *P. tetraurelia* that they also “.*had some success in directly manipulating small organelles such as mitochondria*.” . A few years later, mitochondria from *Physarum polycephalum*, a slime mold, were optically trapped for selection in order to subject the mtDNA to PCR analysis (Kuroiwa et al., 1996). However, mitochondria were trapped with the OT in a microflow channel, after the cells were lysed with a LS, and not in whole living cells. Much later, a similar approach has been employed to select mitochondria for genetic analysis in relation to mtDNA-derived disorders (Reiner et al., 2010). Cells were first tagged with Mitotracker Green, then trapped with an OT and lysed with LS. When the intracellular contents spilled, the OT were switched on to capture the fluorescent mitochondria, which were finally directed toward a micropipette for collection and PCR analysis. Separation of mitochondria by OT has been proved a better approach as compared to laser capture microdissection and flow cytometry (Pflugradt et al., 2011).

Naked molecular DNA has been trapped and studied with OT. Chiu and Zare optically trapped a single DNA molecule from a bacteriophage lambda with a cw 647 nm laser (Chiu and Zare, 1996). The difference here, as compared with many other reports of mechanical properties of DNA, is that the DNA molecule was directly trapped by the OT, without mediation of polystyrene or glass microbeads, so commonly employed to execute OT experiments with DNA (Heller et al., 2014). To achieve direct optical trapping the DNA molecule had to be in a supercoiled state (probably to increase refractive index mismatch with the medium to favor optical trapping), something achieved by tagging the DNA with intercalating YOYO dye in saturating conditions. Later publications reported that it was indeed necessary to achieve a compacted enough state for the DNA molecule to be affected by the OT, highlighting the role of a threshold refractive index mismatch for the optical trap to work properly on DNA (Katsura et al., 1998; Matsuzawa et al., 2002). The technique was further refined to allow for bulk manipulation of intact, long DNA molecules which, thereafter, were microdissected to obtain DNA libraries (Mizuno and Katsura, 2002). The whole Mb-size genome of *Thermococcus kodakaraensis* was manipulated with OT by controlling its structural phase with different solute concentrations (Oana et al., 2005).

**FIGURE 8:**
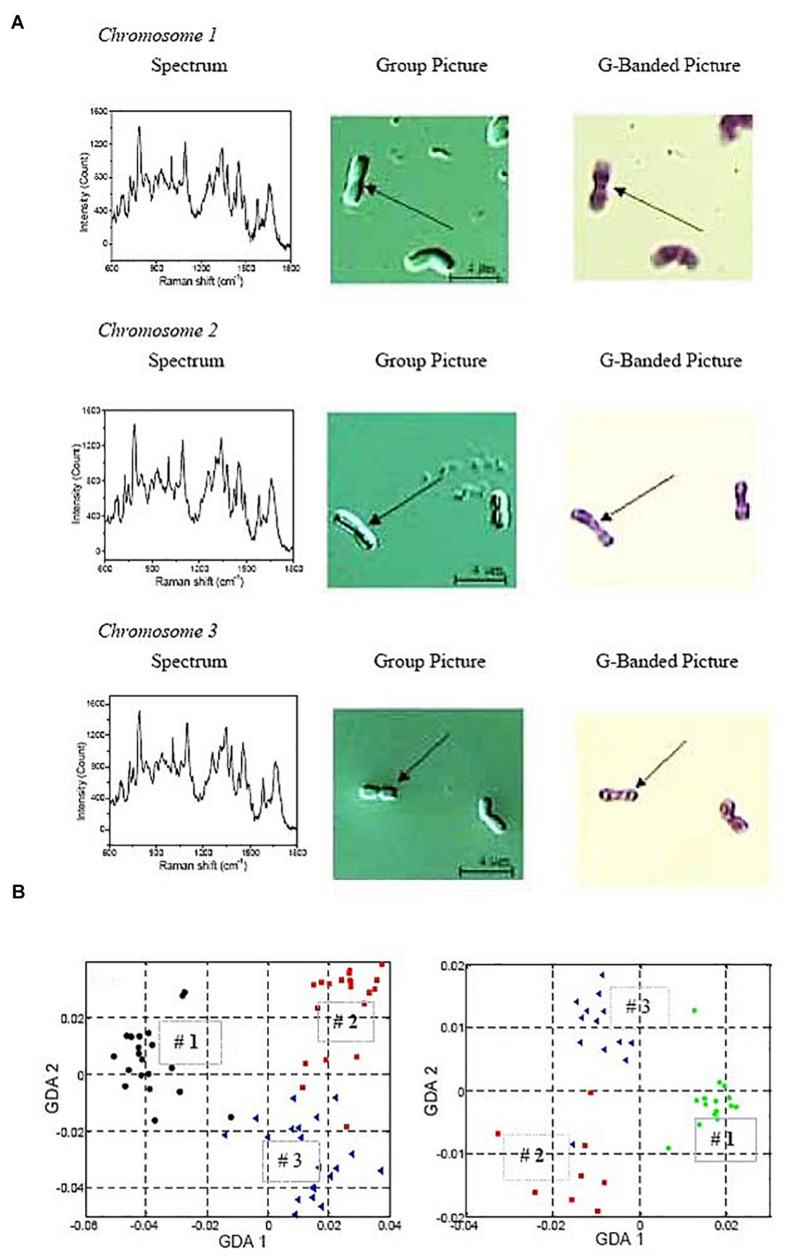
Raman spectrum analysis of optically trapped human chromosomes 1-3. **(A)** Typical Raman spectra (left panels) and G-banding images (right panels) of individual chromosomes. The purpose is to show how the Raman spectrum of an individual chromosome looks like, and how it is related to the positive identification with G-banding. **(B)** Generalized discriminate analysis (GDA) plots of all three chromosome numbers. GDA plot using all data collected from all three chromosomes and normalizing the peaks by a chosen standard inverse wavelength of 783cm^–1^. Chromosome 1 is represented as black circles, chromosome 2 as red squares, and chromosome 3 as blue triangles. On the left, Raman spectra of chromosomes isolated from 6 donors over 12 different days. On the right, Raman spectra from the chromosomes of a single individual over 6 different days. Reproduced with permission from Ojeda et al. (2006).

The optical manipulation of whole DNA molecules has been successfully combined with analytical tools to study their composition and structure. Taking advantage of the mechano-chemical properties of DNA under OT (Hormeño and Arias-Gonzalez, 2006), it has been possible to measure the size of a DNA molecule through the interplay among optical trapping force, hydrodynamic drag and centrifugal force (Hirano et al., 2008). DNA molecules of sizes 50–400 kb were correctly sized using this method. In another application, human chromosomes 1, 2 and 3 from white blood cells were optically trapped in a microchannel and, at the same time, laser-scanned with a second laser to obtain the Raman spectrum of each chromosome (Figure 8; Ojeda et al., 2006). These Raman spectra were compared to Giemsa banding (Figure 8A) and identification patterns, characteristic of each chromosome could be established (Figure 8B). Therefore, it is possible to correctly identify chromosomes through their particular Raman spectrum. For example, chromosomes 1-3 plot stably in different regions of GDA (generalized discriminate analysis) space which allows for robust identification (Figure 8B). This can have applications similar to cellular assessment by flow cytometry in terms of high-throughput cytogenetic analysis.

## Combined LS and OT for Genetic Material Manipulation

Given their respective features and advantages, it is clear that LS and OT were to be combined in a single, powerful platform to undertake complex biophotonic procedures. Since the early 1990s there exist description and reviews on the parallel or sequential use of LS and OT in biological systems (Weber and Greulich, 1992; Ponelies et al., 1994; Berns and Greulich, 2007; Greulich, 2017). If, in addition, these techniques are guided by fluorescence microscopy, a very efficient all-optical setup can be implemented capable of real-time manipulation of living cells or biostructures. Some relevant examples will be presented to show the range of tasks achievable with these approaches.

### Chromosome Manipulation

Combined LS-OT platforms have been widely employed in the study of chromosome and mitotic machinery manipulation, in order to study cell division processes, genomic instability, or repair mechanisms, to cite a few (Berns et al., 2006). One of the first combined use was implemented to assess the behavior of chromosome fragments during mitosis (Liang et al., 1993). Anaphase chromosomes in PTK2 cells were first microablated with LS. Then, the chromosome fragments produced were left to its own or they were trapped with an OT. It was shown that chromosome fragments could be optically trapped. Also, these actively trapped fragments displayed different kinetics (some ended up in the sister cell) from those left undisturbed after the microablation. A similar paper followed shortly, this time employing a different cell model (newt lung cells) (Liang et al., 1994). These cells provided a better cellular test field, with flattened cells and a less “crowded” mitotic apparatus, which allowed for better control and positioning of the chromosome fragments by the OT. More recently, a user-friendly platform has been implemented to provide an easier tool to assess chromosome kinetics in laser-treated cells, incorporating one LS and two OT for finer manipulation (Harsono et al., 2013). Thanks to these methodological approaches it has been possible to provide direct measurements of the forces necessary to move a mammalian chromosome (CHO-K1 cell line) in aqueous solution, accounting for the particle shape and hydrodynamic drag (Khatibzadeh et al., 2014). The measured forces were in the range 0.8–5 pN, which agree with calculated values of 0.1–12 pN for the forces exerted by the mitotic spindle. For recent reviews on this relevant topic the reader can consult Forer et al. (2015) and Berns (2020).

**FIGURE 9:**
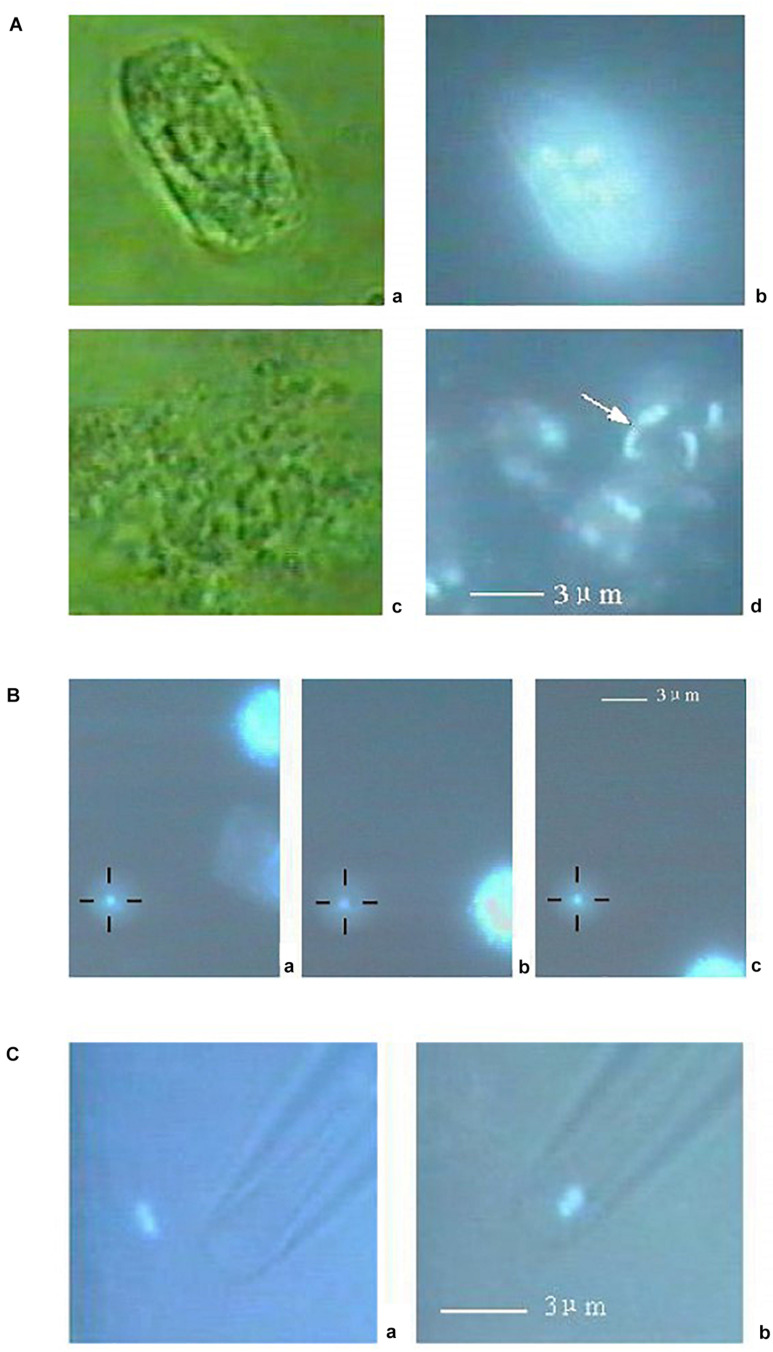
Combined use of LS and OT to obtain individual chromosomes from plant cells. **(A)** A rice root meristem cell (subpanel a) observed by 100 × object lens. Subpanel b: Fluorescence image of the same cell under ultraviolet illumination. There are many bright points, which can be identified as chromosomes, distinct from each other by a clear boundary. Subpanel c: The cell was fragmented by the LS. Subpanel d: Fluorescent image of the same cell crushed by the LS, showing tiny bright rods clearly distinct from each other. The position of a centromere as shown in subpanel d can be noticed on some chromosome as a depressed point (arrow head). **(B)** A chromosome (subpanel a, small bright point) is fixed at the center of the black cross by optical tweezers. Subpanels b and c: The chromosome remains in the center of the black cross, whereas other cellular remnants move away from the area as the stage moves. **(C)** The chromosome (subpanel a, bright point) is near the tip of a capillary. Subpanel b: After the OT is turned off, the chromosome is immediately aspirated into the capillary. The scale bars represent 3 μm. ©IOP Publishing. Reproduced with permission from Wang et al. (2004). All rights reserved.

Two examples will further illustrate the potential of the LS + OT platform for chromosome manipulation. The first one is a publication reporting the isolation of a single chromosome from a rice plant cell (Wang et al., 2004). A suspension of root hair cells was freshly prepared and incubated with DAPI to fluorescently-tag chromatin. A particular cell was selected and lysed with a third harmonic (354 nm) ns pulsed Nd:YAG LS (Figure 9A). Individual chromosomes, displaying blue fluorescence under UV excitation, were spilled into the medium after microablation (Figure 9A subpanel d). One chromosome was selected and trapped with the OT (Figure 9B). Finally, the OT positioned the selected chromosome at the tip of a glass micropipette for suction and PCR analysis (Figure 9C). This is an elegant example of the kind of precise genetic manipulation and analysis accessible with a combination of LS and OT.

**FIGURE 10:**
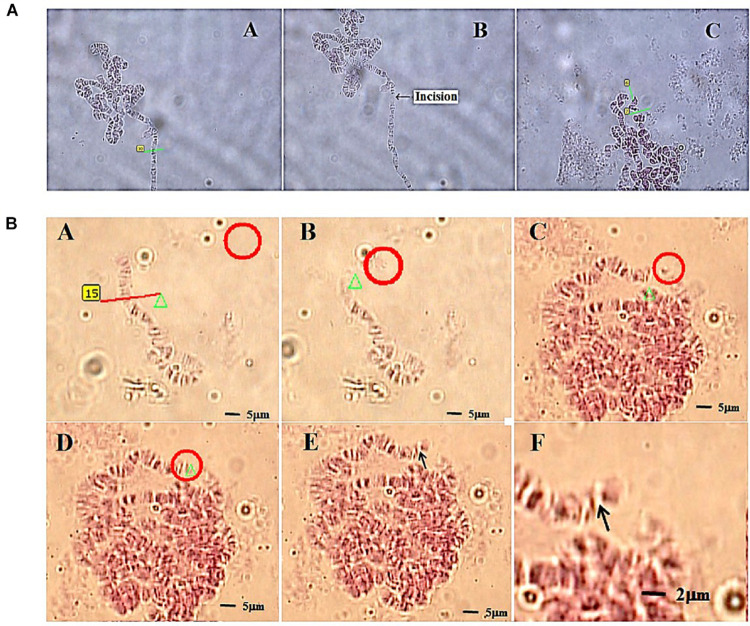
Chromosome welding by use of LS and OT. **(A)** Laser cutting of a polytene chromosome of fruit fly. Subpanel A–A: selecting the incision part on the chromosome before cutting; subpanel A–B: the chromosome after cutting (arrow); subpanel A–C: the cutting on a chromosomal puff (green lines). **(B)** The process of cutting (subpanels B–A, red line), optically trapping (subpanel B–B, red circle) and moving (subpanels B–C, red circle) a chromosome fragment from a short chromosome (subpanel B–A) of a fruit fly, and then welding (subpanel B–D) to a long chromosome of the same fruit fly (red circle). Subpanels B–E and B–F illustrate the chromosome after welding by 63 × and 100 × objectives, respectively. Reproduced with permission from Huang et al. (2018).

The second example, more recent, is quite relevant in the field of genetic manipulation, as it reports on the successful welding of two chromosomes fragments into a single genetic unit (Huang et al., 2018). The chosen biological model were polytene chromosomes of *D. melanogaster*, particularly fitted for manipulation because of their large size. A selected chromosome was first microablated with a 337 nm ns pulsed LS, and a clean cut was operated in one of the chromosome arms (Figure 10A). Then, the chromosome fragment was trapped with a 1064 nm cw OT. Through careful OT manipulation the chromosome fragment was positioned in very close proximity to another, intact chromosome. Employing again the LS, but at a significant lower energy per pulse (111-135 μJ for welding vs. > 270 μJ for cutting), the fragment was “welded” to the intact chromosome (Figure 10B). As will be further discussed in the “PERSPECTIVES” section, this is an important step forward in the manipulation of genetic material, as it paves the way for future genetic modification at the chromosome level. In combination with techniques to be presented in the next section, the possibility to transfer these altered chromosomes, or similar genetic-encoding structures (plasmids or artificial chromosomes), into living cells is very appealing.

### Cell Nanosurgery and Organelle Manipulation

With the LS-OT setup it is possible to execute other kinds of intracellular alterations, like cellular micro- or nanosurgery. One of the first attempts at this was reported by König and collaborators, who successfully ablated chromosomes within living cells with submicrometric accuracy (∼400 nm) (König et al., 1999). Treated cells retained viability for several hours after the procedure, as assessed by calcein-ethidium vital staining. A further advance was reported when subcellular organelles were extracted with OT after the cell was excised with a LS (Shelby et al., 2005). Cells from three different cell lines were subjected to the treatment: CHO, NG108-15 (neuroblastoma-glioma cells) and ES-D3 (murine embryonic stem cells). The LS were based on a 337 nm ns nitrogen laser and the OT on a 1064 nm cw Nd:YAG laser. The cellular incision was carried out with LS energies of 0.5–1 μJ, which led to micrometric (∼1–3 μm) cut. Under these conditions cell viability was preserved in the long term and the damaged plasma membrane resealed in a few minutes. While the “wound” was open it was possible to trap an intracellular organelle with the OT and transport it across the cut into the extracellular space. Trapped and extracted organelles included lysosomes and mitochondria. The fact that mitochondria were successfully moved out a cell seems quite relevant within the scope of this review, as mitochondria carry their own set of genetic material. Mitochondrial genetic alterations are the cause of many cellular alterations and human disorders. Therefore, the possibility to transfer different types of mitochondria among cell targets looks very promising to study or treat some of these conditions (see section “PERSPECTIVES” below).

**FIGURE 11:**
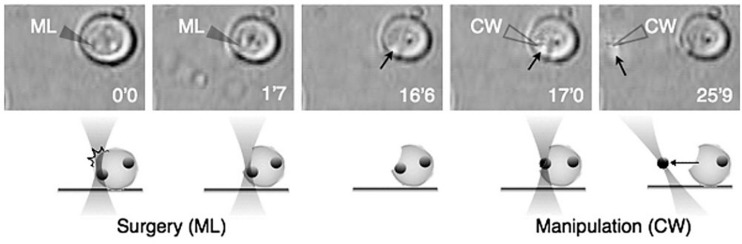
Intracellular organelle extraction **(left images)** and subsequent manipulation **(right images)** using the combined techniques of optical surgery and trapping. Unfilled and filled triangles indicate the position of the laser focus of the cw and femtosecond-pulsed Ti:sapphire laser, respectively. Black arrows indicate targeted intracellular organelle. Reprinted with permission from Ando et al. (2008).

A few years later a similar approach was introduced. However, in this case, the same laser was employed for both the LS and the OT (Ando et al., 2008). A Ti:sapphire fs laser provided the laser light at 780 nm for both systems. The biological model studied were cells of *S. cerevisiae*. When the Ti:sapphire laser was operated in cw (10 mW) the output was used to optically trap the yeast cells. At any time the laser could be switched to mode-locking, thereby emitting fs pulses and operating as a LS (2–10 mW average power). By rapidly switching back and forth between cw and pulsed modes, the laser successfully trapped and cut cells, and extracted internal, but undisclosed, organelles (Figure 11). Finally, Raabe et al. were able to produce enucleated and binucleated cells in a *S. pombe* model using a 405 nm ps LS (Raabe et al., 2009). By ablating the microtubules and mitotic spindle at precise moments during mitosis, one of the daughter cells inherited two nuclei, while its twin receive none. Surprisingly, the enucleated cell was “alive” (capable of sustaining metabolic activity) for several hours after the procedure. This makes an interesting model to study the possible role of mRNA and ncRNA in transiently supporting metabolism without any new nuclear transcription product.

After a survey of relevant literature related to LS, OT and combined LS plus OT for the manipulation of genetic material in diverse scenarios, some potential fields for development and new ideas will be presented in the last section.

## Perspectives

In this final section some discussions and potential developmental lines in the field of optical manipulation of genetic material will be provided. By no means does this summarize the whole development potential of these techniques. This section is intended as a brief overview of interesting extensions of current applications. Some commentaries and ideas try to reinforce research in some areas already in motion, while others are meant as out-of-the-box proposals which could open new avenues in the field.

### Cellular Genetic Engineering

An area that should benefit from future developments is that of cellular surgery (see section “Cell Nanosurgery and Organelle Manipulation”). The possibility to trap, move and extract cellular organelles, including the nucleus or DNA-carrying mitochondria, for example, have been experimentally proved (Shelby et al., 2005; Ando et al., 2008; Raabe et al., 2009). A next step would be to try to introduce a free-standing organelle into another cell (cell transplant). This is not an easy task, of course, but such a kind of cellular transplantation should provide relevant information about cellular processes, particularly if the introduced organelle or genetic-encoding structure has known or artificially introduced mutations of interest. Complementary techniques can be combined with the LS-OT tandem, to facilitate the incorporation of the foreign structure. For example, the so-called “photothermal nanoblade” may help in this task, as it permits fine control of cell surgery (Wu et al., 2010, 2015). In fact, this technique has already been applied to transfer healthy mitochondria to impaired-respiration cells with defective mitochondria (Wu et al., 2016). Very recently, a methodology for the successful transfer of individual mitochondria between donor and acceptor cells has been developed (Shakor et al., 2019). The method relies on glass micropipettes and a robotized stage to achieve the transfer. However, given the increasing spatial-temporal control in LS-OT setups (Yang et al., 2017; Sitnikov et al., 2018), similar results should be seen in the near future employing these setups. This successful methodology for mitochondrial transfer can have wide implications for the profiling and therapeutics of a whole family of diseases related to dysfunctional mitochondria (e.g., Huntington’s disease, mitochondrial myopathies, mtDNA depletion syndrome, or cancer). Many of these disorders have a cause in the mtDNA and its defects, thus a potential “healthy mitochondrial transplant” could be a real treatment in the near future.

### Chromatin and Chromosomes Lesions

One of the most successful applications of LS-OT has been the trapping and/or damaging of (mitotic) chromosomes. This kind of studies will keep providing critical information on mitosis kinetics, regulation and consequences of chromosomal dysfunctionality (Kong et al., 2017; Milas et al., 2018; Odell et al., 2019). For example, a very recent publication reports on a very fine level of LS disruption of particular mitotic machinery elements in order to better understand mitotic chromosome migration (Forer and Berns, 2020). The LS were employed to first disrupt chromosomal arms, involved in inter-chromosomic tethers connecting oppositely migrating homologous chromosomes, and then damage the kinetochore spindle fibers on individual chromosomes. This procedure has determined that inter-chromosomic tethers regulate the chromosome migration in anaphase in an insect biological model. This kind of approach can have important repercussions in models of perturbed chromosomal migration, fundamental to understand cytogenetic mutations and related disorders (chromosomal abnormalities, cancer, etc.). Additionally, it has been recently highlighted the importance of the DDR activated during mitosis by the selective LS-induced damage to the chromosomes (Gomez-Godinez et al., 2010, 2020). Noteworthy, the degree of mitotic blocking/delaying depends on which mitotic stage the cell is (e.g., metaphase vs. anaphase) and what chromosome part is damaged (e.g., central vs. distal) (Baker et al., 2010). In view of the results obtained, the telomeres, the most distal structures in the chromosome, are to be considered DDR-hotspots. Damage or alteration of their structure-function seems to trigger a robust DDR (Alcaraz Silva et al., 2013, 2014; Hustedt and Durocher, 2016). Given the role of telomeres in processes such as aging, genome preservation/instability and cancer, the LS-OT manipulation of telomeres should be a particularly intense research area. Additionally, the recently reported possibility to conduct chromosomal welding represents an excellent proxy for a plethora of chromosomal structural mutations and fusion chromosomes (Huang et al., 2018).

### Micronuclei and Chromothripsis Induction

An intense area of research in Genetics, Cytogenetics and Cancer Biology is the recently described phenomenon of chromothripsis (Ly and Cleveland, 2017; Umbreit et al., 2020). Chromothripsis is a process in which a mitotic chromosome is inadequately attached to the mitotic spindle and remains adrift after telophase. It develops its own nuclear envelope and becomes what is commonly known as a micronucleus. In a second round of division, the micronucleus is detected and an inadequate genetic damage repair response ensues which, in fact, defectively identifies the rogue chromosome and proceeds to break it into many fragments. These fragments can end being reattached to other chromosomes, forming circular chromatin elements or being degraded altogether. The end result is a massive chromosomal rearrangement which can result in some genes being completely lost, being located in incorrect chromosomes, and/or get replicated tens or even hundreds of times (Zhang et al., 2015). This has dramatic consequences for the genomic stability of the affected cells, and is, at present, considered one of the principal cytogenetic mechanisms behind tumoral cell induction and progression (Ly and Cleveland, 2017).

Given the fine control that OT offer to trap and manipulate either whole chromosomes or chromosome fragments (Berns et al., 1989; Harsono et al., 2013; Milas et al., 2018), plus the possibility to directly produce chromosome fragments with LS (Berns et al., 2006), it is clear that these tools should be promptly employed to discern the mechanisms of chromothripsis. For example, less subtle procedures, like exposure of cell cultures to mild concentrations of ROS, has been proved to be an effective method to induce large amounts of micronuclei (Blázquez-Castro and Stockert, 2015). As discussed above (see section “Mechanisms of Action of LS”) LS can be an efficient source of ROS and reactive chemistry over submicrometric volumes. Therefore, LS should be able to induce chromothripsis either directly, by severing a chromosome fragment, or indirectly, by producing ROS in close proximity to the nucleus/chromosomes.

In regards to OT use in connection to chromothripsis there are some alternatives to study the process. In spontaneous chromothripsis a biomechanical perturbation is at the initiation of the process (Booth et al., 2019; Bennabi et al., 2020; Liu and Pellman, 2020). This is precisely the kind of perturbation (mechanical) an OT can provide with accuracy. Oxidative stress brought about by excessive ROS is a recognized driving agent for micronuclei production and genomic instability induction (Xu et al., 2014; Guo et al., 2019). Certain wavelengths (discussed in section “Mechanisms of Damage in OT”) can directly produce ^1^O_2_, which should produce similar outcomes to those mentioned above for LS in order to induce chromosomal damage. Particularly appealing is the possibility to systematically study which chromosomes are more prone to undergo chromothripsis, as the tandem LS-OT permits selection of particular chromosome targets (perhaps identified morphologically and/or by FISH). As previously mentioned, telomeres can be selectively perturbed with LS. Telomeres are critical structures in the development of chromothripsis (Aguilera and García-Muse, 2013), thus opening another research avenue for this critical cytogenetic process (Baker et al., 2010). There are reports from long ago on the production of micronuclei by means of LS-OT (Brenner et al., 1980; McNeill and Berns, 1981; Hu et al., 1989). This means the process is feasible. Hence, the study of chromothripsis induction and its consequences by LS, OT and LS-OT seems a matter of fine-tuning the process and assessing cell genomic stability on a longer term.

### Redox Genetics and Epigenetics

As mentioned in the section “Mechanisms of Damage in OT,” certain wavelengths commonly employed for OT can directly excite dissolved O_2_ to reactive ^1^O_2_ in the biological medium where irradiation experiments are proceeding (Blázquez-Castro, 2017, 2019). What is a drawback for OT use can be an asset for studying redox responses in a very similar setup (Blázquez-Castro et al., 2020). The exposure conditions are much closer to those typical of OT, low intensity pulsed or cw laser emission, rather than LS. In recent years, the importance of redox signaling in cell biology (Carrasco et al., 2016), and genetic and epigenetic processes has been increasing (Kreuz and Fischle, 2016). For example, it has been reported that mitochondria cluster around the nucleus to provide ROS, favoring an oxidative environment to orchestrate HIF-1 expression (Al-Mehdi et al., 2012). This is partly due to the production of oxidized DNA bases (mainly 8-oxoG) which do act as epigenetic markers for genetic expression rather than as damaged biomolecules (Di Mascio et al., 2019; Epe, 2020). In the same line, the physiologically expressed lysine-specific histone demethylase 1A (LSD-1) has been shown to purposefully oxidize DNA bases to signal for genetic expression (Epe, 2020). Interestingly, LSD-1 has already been induced by a LS-OT treatment, as recently reported in connection to DDR induction (Duquette et al., 2018). Milder laser microirradiation treatments have already proved that it is possible to grossly modulate the cellular cell cycle through laser exposure to NIR (760–765 nm) wavelengths (Blázquez-Castro et al., 2018a). Therefore, by judiciously choosing the laser parameters (wavelength, pulse duration, power, etc.) it is possible to carry out relevant research on redox cell modulation with OT-like setups (Westberg et al., 2016; Linz et al., 2016; Schmalz et al., 2018; Gomez-Godinez et al., 2020).

### Mechanotransduction

The field of mechanobiology and mechanotransduction in cells is experiencing a renewed interest at present (Yusko and Asbury, 2014; Mathieu and Manneville, 2019; Moujaber and Stochaj, 2020). The field studies the cellular mechanisms to sense and respond to different types of forces acting upon cells. Force transduction has been described as acting at two levels: biochemical transduction by mechano-sensitive channel proteins and direct mechano-modulation of the nucleus by relaxing or compressing chromatin domains (Liu, 2016; Trubelja and Bao, 2018). Mechanotransduction is shown to be very relevant in such processes as cell migration, cell division or tissue regeneration, and it is at the origin of several disorders (Isermann and Lammerding, 2013). With their capability to exert forces at precise levels and microscopic locations, OT make a perfect candidate to move from a relatively passive role as a cell “holder” to a more active interaction to assess mechanotransducing pathways (Botvinick and Wang, 2007; Liu, 2016). An immediate field of application in relation to genetics and cytogenetics could be the study of nuclear and chromatin responses to mechanical cues induced by OT (Haase et al., 2016; Guo et al., 2018; Lele et al., 2018), and assess the mechanisms of mechano-epigenetics engagement (Missirlis, 2016).

### Photothermal Modulation

In line with the previous research proposal, it should be possible to also study cellular mechano-responses taking advantage of the photothermal/thermomechanical response to certain patterns of microirradiation. Making use of NIR wavelengths absorbed by water the whole cell, or certain subcellular structures, can be selectively heated. By establishing an intermittent irradiation of the region of interest, or periodically exciting it with a scanning mode, cycles of thermally driven expansions and contractions can be obtained. This can induce mechanotransduction, as mentioned above. In this line, a research group found that cells subjected to particular vibrational frequencies in the range 10–1,000 Hz displayed a severely increased cell death (Ng et al., 2013; Sun et al., 2017). The vibrations were mechanically produced, but adequate intermittent microirradiation with NIR light could lead to similar results. It has been proposed to achieve a selective photothermal cancer therapy taking advantage of the slightly different thermomechanical properties of normal vs. tumoral cells (Letfullin and Szatkowski, 2017). In parallel, it has been experimentally proved that the isolated nuclei of cells present a non-linear expansion-contraction behavior under different photothermal conditions (Chan et al., 2017). These works, although preliminary, suggest that there is a rich potential for a photothermal-OT approach, to be developed within a mechanotransduction signaling paradigm.

### Transversal Technical Approaches

Last, LS and OT can be combined with other established or emerging techniques. Indirect OT approaches have proven very convenient when biological samples display inadequate optical or mechanical properties that make direct optical trapping inappropriate (Gerena et al., 2019). In this sense, tailored micro- and nano-machines can be trapped with OT and then made to interact with biological structures (Andrew et al., 2020). This is an example of indirect manipulation of a biological object by OT. Torques have been applied to cells with these indirect methods. As to sample analysis, OT can be combined with optical analytical tools as was shown, for example, with Raman spectra of human chromosomes (Figure 8; Ojeda et al., 2006). The fact that OT use a laser as the light source simplifies such measurement efforts, as many efficient analytical methods employ lasers to obtain relevant data (fluorescence microscopy, Raman spectroscopy, flow cytometry, etc.). Recently, the IR nanospectroscopic mapping of a metaphase chromosome has been reported (Lipiec et al., 2019). These combined techniques, OT plus optical analysis, can provide the basis for high-speed automatized chromosomal identification without relying to classical staining (e.g., Giemsa).

Optical trapping becomes less robust when approaching target sizes below 100–200 nm (Bradac, 2018). Plasmonics can help with this issue as the phenomenon greatly amplifies electric fields (from the OT, for example) precisely at the < 100 nm scale (Boulais et al., 2013; Hu et al., 2020). This can enhance trapping forces and nanoablation at the nanometric scale, while still employing optical excitation (Csaki et al., 2007; Boulais et al., 2013). An alternative approach to produce and enhance electric fields with microirradiation is to use photoresponsive ferroelectrics. Several of these materials display the so called bulk photovoltaic effect and produce enormous electric fields in the near-field when light-excited. A combination of OT and electrophoresis/dielectrophoresis by photoactivated ferroelectrics can result in complex or massive trapping patterns (García-Cabañes et al., 2018). Living cells perturbation has been already proved with this microirradiation approach (Blázquez-Castro et al., 2011). Recently, a review on this and many other applications of ferroelectrics in Biology has been published (Blázquez-Castro et al., 2018b). It would be interesting to combine OT with photoresponsive ferroelectrics for Cell Biology and Genetics, particularly when these materials have been shown capable of driving controlled movement of micrometric liquid volumes, relevant when talking about cellular processes (Nasti et al., 2020).

## Conclusion

Laser scissors and OT provide very relevant tools for study and understanding in the fields of Genetics, Cytogenetics and Cell Biology. In parallel, these tools have provided very high levels of control and manipulation over particular genetic molecules and structures carrying genetic information, opening new avenues and applications in the field. We feel that the possibilities these methodologies have to offer in the near future will be even more fascinating. It is our hope that we have been able to transmit the critical contribution of LS and OT to the field, and stirred the interest and curiosity of the research community to develop further applications from new angles.

## Author Contributions

AB-C prepared the outline, wrote and edited the manuscript, and prepared figures and tables. JS wrote and edited the manuscript. JF-P edited and revised the manuscript. All authors approved the manuscript for publication.

## Conflict of Interest

The authors declare that the research was conducted in the absence of any commercial or financial relationships that could be construed as a potential conflict of interest.
